# Context-dependent similarity analysis of analogue series for structure–activity relationship transfer based on a concept from natural language processing

**DOI:** 10.1186/s13321-025-00951-3

**Published:** 2025-01-15

**Authors:** Atsushi Yoshimori, Jürgen Bajorath

**Affiliations:** 1 Institute for Theoretical Medicine, Inc., 26-1 Muraoka-Higashi 2-chome, Fujisawa, Kanagawa 251-0012 Japan; 2 Department of Life Science Informatics and Data Science, B-IT, LIMES Program Unit Chemical Biology and Medicinal Chemistry, University of Bonn, Friedrich-Hirzebruch-Allee 5/6, 53115 Bonn, Germany; 3 Lamarr Institute for Machine Learning and Artificial Intelligence, University of Bonn, Friedrich-Hirzebruch-Allee 5/6, 53115 Bonn, Germany

**Keywords:** Active analogue series, Potency progression, Series alignment, SAR transfer, Fragment pair relationships, Context-dependent similarity

## Abstract

Analogue series (AS) are generated during compound optimization in medicinal chemistry and are the major source of structure–activity relationship (SAR) information. Pairs of active AS consisting of compounds with corresponding substituents and comparable potency progression represent SAR transfer events for the same target or across different targets. We report a new computational approach to systematically search for SAR transfer series that combines an AS alignment algorithm with context-depending similarity assessment based on vector embeddings adapted from natural language processing. The methodology comprehensively accounts for substituent similarity, identifies non-classical bioisosteres, captures substituent-property relationships, and generates accurate AS alignments. Context-dependent similarity assessment is conceptually novel in computational medicinal chemistry and should also be of interest for other applications.

**Scientific contribution**

A method is reported to systematically search for and align analogue series with SAR transfer potential. Central to the approach is the assessment of context-dependent similarity for substituents, a new concept in cheminformatics, which is based upon vector embeddings and word pair relationships adapted from natural language processing.

## Introduction

The exploration of structure–activity relationships (SARs) of small molecules is a major task in medicinal chemistry, providing the basis for compound optimization [1–6]. SARs are typically explored in a target-dependent manner by generating structural analogues of active compounds. Accordingly, analogue series (AS) represent the primary source of SAR information in the practice of medicinal chemistry [6–9]. While AS are typically generated on a case-by-case basis in the context of hit-to-lead or lead optimization projects, they can also be algorithmically extracted from large compound data sets originating from medicinal chemistry [9–11].

In compound optimization, an important question is whether different AS might display similar SAR trends. For instance, if an AS with sustainable SARs and desirable potency progression might be liable due to unfavorable in vitro or in vivo properties, one would like to replace this series with another having comparable SAR characteristics, which is referred to as SAR transfer [12]. Pairs of AS that contain different core structures and analogue pairs with corresponding substitution patterns and show comparable potency progression represent SAR transfer incidents [12]. For many pharmaceutical targets, such SAR transfer series have been identified computationally by searching for AS with corresponding analogues, potency-based ordering of analogues, and comparison of the potency gradients [12, 13]. Target-based SAR transfer has also been explored using  X-ray structures of complexes of individual target proteins with different analogues [14].

Importantly, SAR transfer events captured by qualifying AS pairs might also involve different targets [15]. SAR transfer across different targets at least in part mirrors generally applied medicinal chemistry strategies to optimize ligand-target interactions, for example, the use of hydrophobic substituents of increasing size to “fill” hydrophobic binding pockets. To systematically search for SAR transfer across different targets, we previously introduced a computational methodology to search for AS with SAR transfer potential and find the best possible alignment meeting SAR transfer criteria [15]. The approach relied on the quantification of substituent (fragment) similarity in combination with dynamic programming to align AS analogously to amino acid or nucleotide sequences. Applying this AS alignment approach, SAR transfer events involving different targets were frequently detected [15].

Herein, we report a method for the systematic identification of SAR transfer events and compound design that is based on similarity concepts from language processing (NLP).

## Methods

### Identification of analogue series

Compounds with available IC_50_ values (standard relation “ = ”) and highest assay confidence score of 9 were extracted from ChEMBL [16] (release 29) and divided into 2240 target-based activity classes. Compounds from each activity class that originated from the same publication were subjected to systematic fragmentation of exocyclic single bonds using the matched molecular pair (MMP) algorithm by Hussain and Rea [10]. The fragmentation was performed using “rdMMPA.FragmentMol” function of RDKit [17]. The input parameters “minCuts”, “maxCuts”, and “maxCutBonds” were set to 1, 1, and 20, respectively. Compound fragmentation produced key (core structure) and value (substituent) fragments from input compounds. Following MMP fragmentation for AS [10, 11], a value fragment was permitted to consist of up to 12 non-hydrogen atoms and a maximum of 30% of the non-hydrogen atoms of the source compound. AS were defined as series of three or more compounds having the same key and different value fragments. Since each identified AS originated from the same publication, assembly of AS combining compounds from different sources was avoided. AS meeting the specified criteria were systematically extracted from all activity classes, yielding a total of 113,113 AS for 2240 target proteins containing 26,795 different value fragments (substituents). All AS were ordered according to increasing compound potency values.

### Fragment representation and similarity

**Fig. 1:**
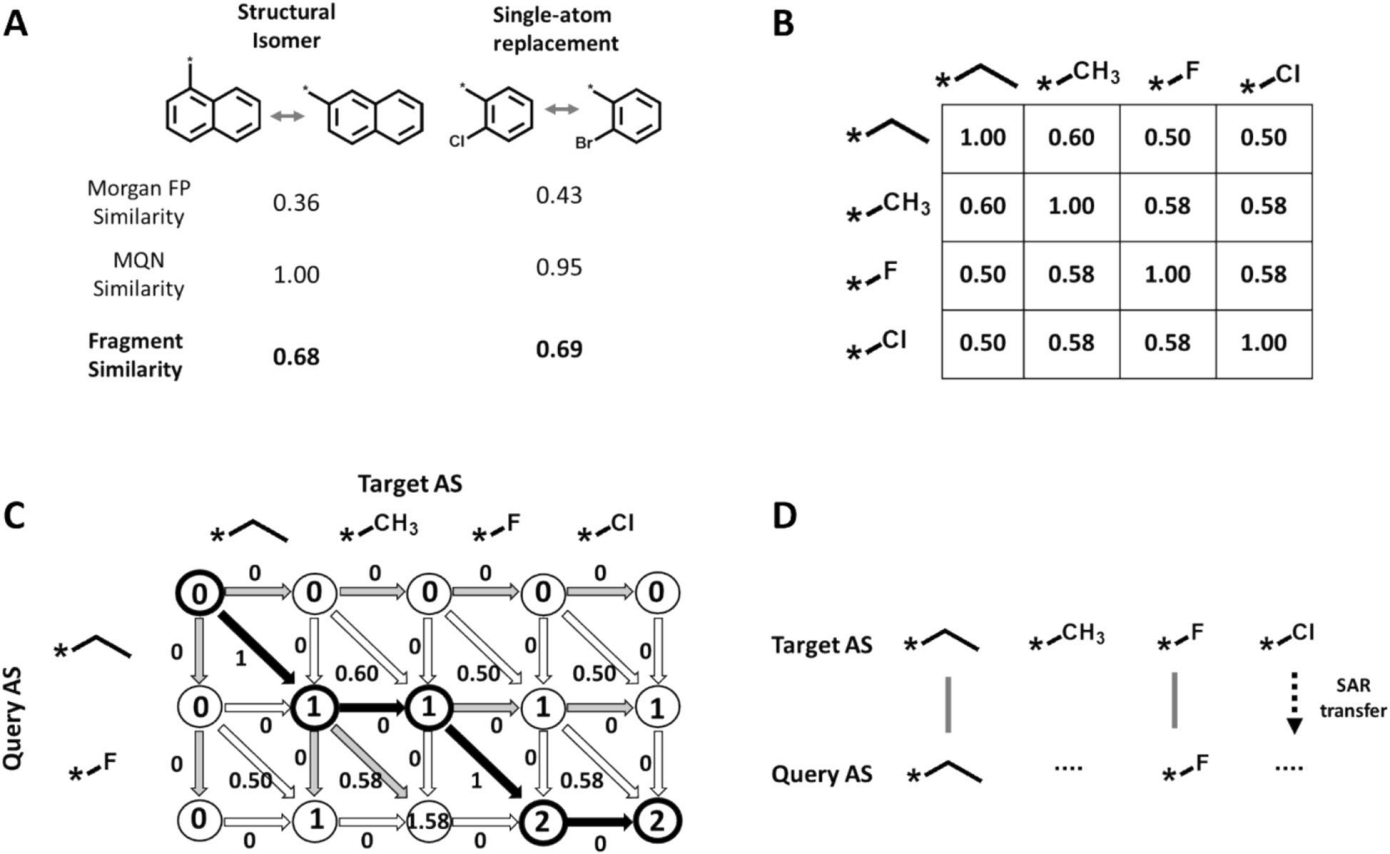
Components of analogue series alignment. **A** Similarity values for exemplary pairs of fragments. Morgan FP Tanimoto, MQN, and combined fragment similarity values for reported. **B** CFR similarity matrix for exemplary substituents (value fragments). **C** Alignment procedure for a query and target AS. The final alignment is obtained based on the highest scoring path in the alignment grid (depicted by the black arrows) obtained by forward and traceback dynamic programming steps. **D** Final alignment of the query and target AS. The dashed arrow indicates a possible SAR transfer analogue for the query AS. The vertical solid gray and horizontal dotted lines indicate exact matches and gaps, respectively. The figure was adapted from reference 15 and modified

Value fragments were represented using the Morgan fingerprint (FP) [18] and molecular quantum number (MQN) descriptors [19]. The combined Morgan FP and MQN descriptor representation was termed conventional fragment representation (CFR). A folded Morgan FP was generated using “AllChem.GetMorganFingerprintAsBitVect” function of RDKit. The input parameters “radius” and “*nBits*” were set to 2 and 1024, respectively. MQN descriptors define a 42-dimensional property space in which each dimension is represented by an atom, bond, or chemical group descriptor with different chemical characteristics [20]. For a compound, the values of these descriptors are calculated from its structure and recorded in a vector. The MQN descriptors were generated using “rdMolDescriptors.MQNs” function of RDKit. Morgan FP similarity was quantified by calculating the Tanimoto coefficient^20^ and MQN similarity (${S}_{ij}^{MQN}$) was defined as:

$${S}_{ij}^{MQN}=\frac{1}{\left(1+\frac{1}{42}{\sum }_{l=1}^{42}\left|{MQN}_{l}^{i}-{MQN}_{l}^{j}\right|\right)}$$

where ${MQN}^{i}$ and ${MQN}^{j}$ are MQN descriptors of the i-th and j-th fragment, respectively. For both measures, the maximal similarity value is 1 and the final CFR similarity was obtained by averaging FP Tanimoto and MQN similarity, thereby combining structural and property similarity specifically for the application to molecular fragments [15]. Figure 1A shows similarity values for exemplary fragment pairs.

### Analogue series alignment

In our previous approach [15], AS with different core structures were aligned using the Needleman-Wunsch dynamic programing algorithm that is typically applied to biological (amino acid or nucleotide) sequences [21]. AS alignments were generated based on CFR similarity of substituents. Initially, all value fragments occurring in AS were sampled and a global CFR similarity matrix was constructed, as illustrated using a small matrix in Fig. 1B. Then, an alignment grid was constructed for a query and target AS, shown Fig. 1C. In the alignment grid, a score $D\left(i,j\right)$ indexed by fragments from each AS was calculated:

$$D\left(i,j\right)=max\left\{\begin{array}{c}D\left(i-1,j-1\right)+s\left({q}_{i},{t}_{j}\right)\\ D\left(i-1,j\right)-gap\\ D\left(i,j-1\right)-gap\end{array}\right.$$

where ${q}_{i}$ is the i-th fragment of the query AS (length n), ${t}_{j}$ is j-th fragment of the target AS (length m), $s\left({q}_{i},{t}_{j}\right)$ is similarity between ${q}_{i}$ and ${t}_{j}$, and $gap$ presents the gap penalty. Here, both $D\left(\text{0,0}\right)$ and gap penalty were consistently set to zero, given that AS are short compared to biological sequences. In the forward step of dynamic programming, grid scores were assigned and their origins recorded. Then, in the traceback dynamic programming step, the highest scoring path connecting grid position $D\left(n,m\right)$ to the origin $D\left(\text{0,0}\right)$ was determined (Fig. 1C). This path yielded the final AS alignment, illustrated in Fig. 1D, with a score normalized by AS length to the value range [0, 1].

### SAR transfer analysis

AS alignments can be used to capture SAR transfer events, that is, detect AS with activity against different targets and corresponding potency progression. Therefore, a key feature of the AS alignment methodology is that it only depends on the assessment of substituent similarity. Accordingly, it is readily applicable to AS containing different core structures. Moreover, AS alignments can be used to predict potent analogues for a query AS by systematically searching for target AS representing SAR transfer events. In Fig. 1D, the dashed arrow on the right indicates a so-called *SAR transfer analogue* in a target sequence that can be used to extend the query sequence with an analogue likely to have further increased potency.

**Fig. 2:**
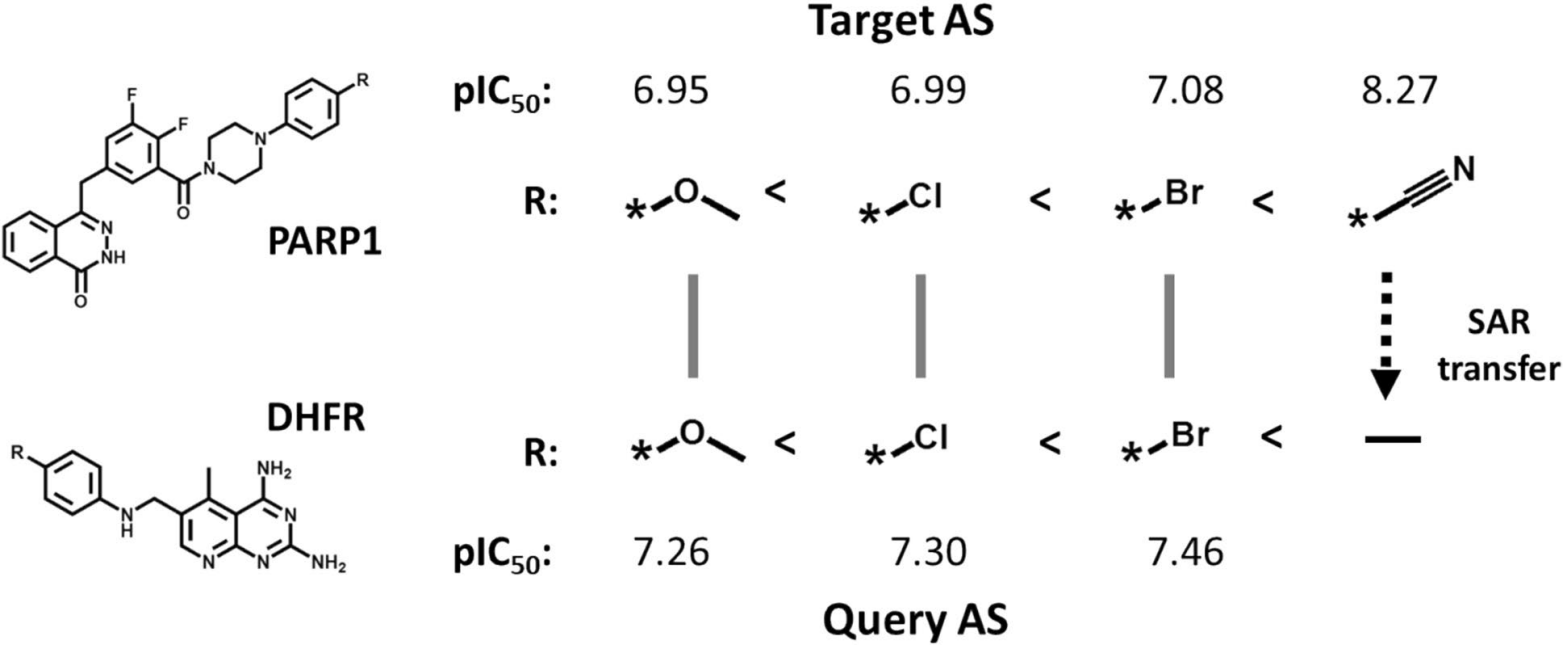
Analogue series alignment-based SAR transfer analysis. An alignment of two exemplary AS with corresponding pairs of analogues and activity against poly[ADP-ribose] polymerase 1 (PARP-1) (Target AS) and dihydrofolate reductase (DHFR) (Query AS) is shown. The dashed arrow indicates a possible SAR transfer analogue for the query AS. The figure was adapted from reference 15 and modified

For the prediction of SAR transfer analogues, a potency-ordered query AS of length *n* can be searched against an AS database for target AS of length *n* + *k* to identify potential SAR transfer alignments enabling analogue transfer predictions. An example of a highest-scoring alignment from a database search capturing an SAR transfer event and enabling analogue prediction is shown in Fig. 2.

### Embedded fragment vector and similarity

**Fig. 3:**
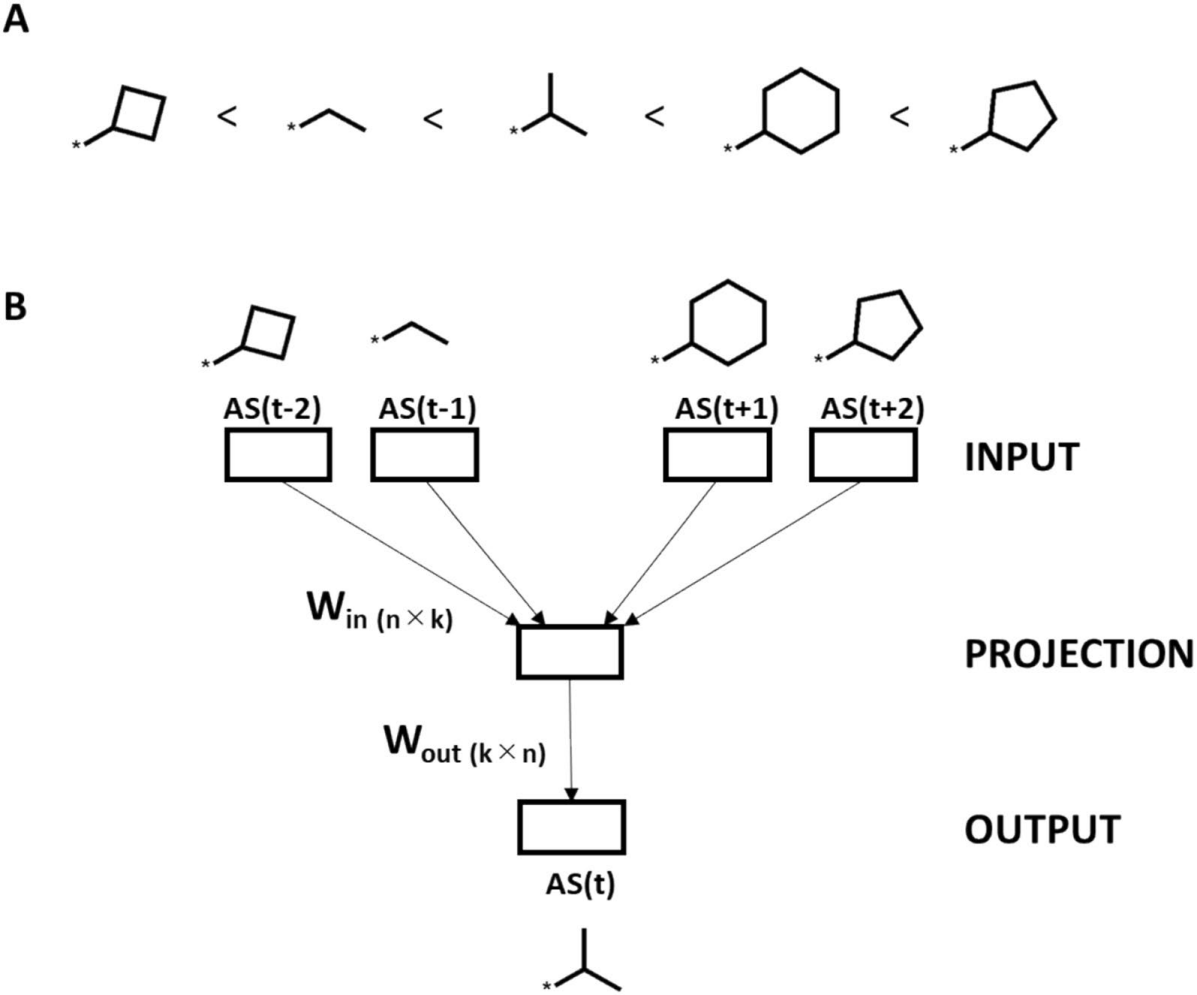
Embedded fragment vector generation. The construction of an EFV via Word2vec is illustrated. In **A**, an exemplary training AS is shown, represented as a sequence of substituents ordered by increasing potency ( <) of the respective analogues. **B** schematically illustrates the architecture of CBOW_W2V for EFV construction consisting of four input layers (with window size of 2), a projection, and an output layer. W_in_ and W_out_ are weights of the model where *n* denotes the size of the vocabulary (corresponding to the different fragments in the AS) and *k* the dimensionality (size) of the EFV. Indices (*t* ± *1/2)* indicate the position of an input fragment/substituent in an AS with respect to the prediction target at position *t*

In NLP [22], Word2vec is a neural network-based methodology for assessing context-dependent similarity of words [23]. Using large numbers of words (or sentences) as input, Word2vec models are derived to generate a high-dimensional vector space and compute continuous vector representations of words such that similar words and words occurring in similar contexts are represented by embedded word vectors that are proximal in space [23]. We used the continuous bag of words (CBOW) variant of Word2vec that predicts words based on surrounding words in vector space, corresponding to preceding and following words in a sequence/sentence [24]. Hence, a CBOW Word2vec (CBOW_W2V) model predicts words in a given vector space context, which is facilitated through the generation of embedded word vectors, corresponding to embedded fragment vectors (EFVs) in our adaptation. By analogy, value fragments (substituents) and AS correspond to “words” and “sentences”, respectively. Figure 3A shows an exemplary training AS (sentence) composed of a sequence of values fragments (words). Figure 3B depicts an exemplary CBOW_W2V model comprising a neural network with four input layers, a single hidden (projection) layer, and an output layer. W_in(n×k)_ and W_out(k×n)_ are weights of the model, where *n* is the size of the vocabulary (equivalent to number of fragment types) and *k* the size of the hidden layer (equivalent to the dimensionality of an EFV). Input fragments are represented as one-hot encoded vectors. The input of the projection layer consists of the mean vector of the individual one-hot encoded fragment vectors multiplied with W_in(n×k)_. As a result of training the model, the EFV of each fragment is obtained from the weight W_in(n×k)_.

The CBOW_W2V model was constructed using “word2vec.Word2Vec” function of *Gensim* [25], a Python library for unsupervised topic modeling, document indexing, retrieval by similarity, and other NLP functionalities. The input parameters “vector_size”, “window”, “min_count”, “sg”, “seed”, and “workers” were set to 100, 5, 1, 0, 8, and 1, respectively. As the input “sentence”, the data set consisting of 113,113 AS was used. The vocabulary (number of fragment types) for the AS data set comprised 26,795 unique substituents. The length of AS and corresponding substituent sequences used for training is flexible and ranged here from three to 322 substituents. After training, the word vector, corresponding to EFV, was generated via the “model.mv” function. For unique substituents, pairwise EFV similarity (${S}_{ij}^{EFV}$) was quantified by calculating cosine similarity [26] defined as:

$${S}_{ij}^{EFV}=\frac{{EFV}^{i}\cdot {EFV}^{j}}{\Vert {EFV}^{i}\Vert \Vert {EFV}^{j}\Vert }$$

where ${EFV}^{i}$ and ${EFV}^{j}$ represent the EFV of the i-th and j-th fragments, respectively.

### Molecular grid map

Molecular grid map (MGM) is a representation projecting a chemical feature space onto a two-dimensional (2D) grid such that decreasing distance between grid points indicated increasing similarity and increasing distance indicated dissimilarity of chemical entities [27]. By assigning property values to grid points, the distribution of molecular properties across data sets can be visualized, providing a global “bird’s eye” view. Herein, fragment (substituent) distributions were analyzed using MGMs. For MGM generation, the 26,795 unique substituents were represented as EFVs of size 100 and the resulting EFV space was subjected to two-step dimension reduction. Initially, principal component analysis (PCA) was carried out to obtain the top-10 principal components (PCs). Following this pre-processing step, t-distributed stochastic neighbor embedding (t-SNE) [28] was performed on the PC space for 3000 iterations with an initial random seed and t-SNE parameter settings n_components = 2 and perplexity = 10, resulting in the 2D projection of fragment space. PCA and t-SNE were carried out using scikit-learn [29]. Each data point in the 2D projection was then mapped onto a regular grid by using the Jonker−Volgenant (J−V) algorithm to solve the associated linear assignment problem [30]. For the fragment data set (including 100 dummy fragments), the size of the resulting MGM was 165 × 163 grid points (cells) onto which different fragment properties calculated with RDKit were mapped.

## Results and discussion

### Fragment similarity and analogue series alignments

In AS alignment, the assessment of substituent similarity plays a critically important role. Since many substituents are small fragments, descriptor-based similarity might not always sufficiently discern fragment similarity relationships to arrive at non-ambiguous AS alignments. Therefore, CFR similarity was originally defined to include the comparison of topological patterns and other chemical properties and thus account for fragment similarity relationships at a high chemical “resolution” [15]. However, we have reasoned that perceiving AS as “sentences” and evaluating substituent (”word”) similarity in a context-dependent manner might make it possible to approach the AS alignment task in a methodologically distinct manner. Specifically, EFVs are used to establish context-dependent word pair relationships [23]. For instance, the relationship “(Paris – France) + Italy = Rome” that ultimately associates the word “Rome” with “Italy”, is established by subtracting the EFVs of the words “Paris” and “France” and adding the result to another word, “Italy”, to provide the context for the prediction of “Rome” [23]. Hence, “Rome” was predicted from the relationship between the three preceding words. In our adaptation, we follow this concept and use three fragments for context-dependent prediction. However, this scheme can be modified, depending on the application, and other queries can be derived. As another example, a chemical word pair context can be established, for instance, by EFV operations associating element names with corresponding standard abbreviations: (Cu – copper) + zinc = Zn. The generation of such word pair relationship contexts was thought to have high potential for assessing substituent similarity in pairs of AS, thus motivating our adaptation of this NLP concept for AS alignment, as discussed in the following.

### Searching for bioisosteres with query fragments

As a pilot application for the approach and its comparison with the original CFR similarity-based methodology, we investigated similarity searching for bioisosteres, which are defined as alternative substituents or groups that are structurally related and have similar physicochemical characteristics and protein-ligand interaction potential [31]. Therefore, in compound optimization, bioisosteric replacements are expected produce analogues retaining the biological activity of a given lead compound [31]. Accordingly, bioisosteres represent alternative substituents that are most likely to conserve biological activities of AS. Hence, they represent an attractive test case for evaluation of alternative similarity measures. Viewed from an NLP perspective, words representing bioisosteres are expected to share latent semantic features (that is, chemical and biological properties) a predictive model is challenged to learn in order to derive relationships between words in similar contexts.

In seminal work, Burger distinguished between classical and non-classical bioisosteres [32]. Classical bioisosteres are composed of atoms or groups having the same valence and ring equivalents. Examples of monovalent classical bioisosteres include chloro and bromo substituents or the hydroxyl and thiol groups. Non-classical bioisosteres, on the other hand, are typically composed of different numbers of atoms and might have varying electronic and/or steric properties such as, for example, the carboxyl group and tetrazole ring [31, 32].

**Fig. 4:**
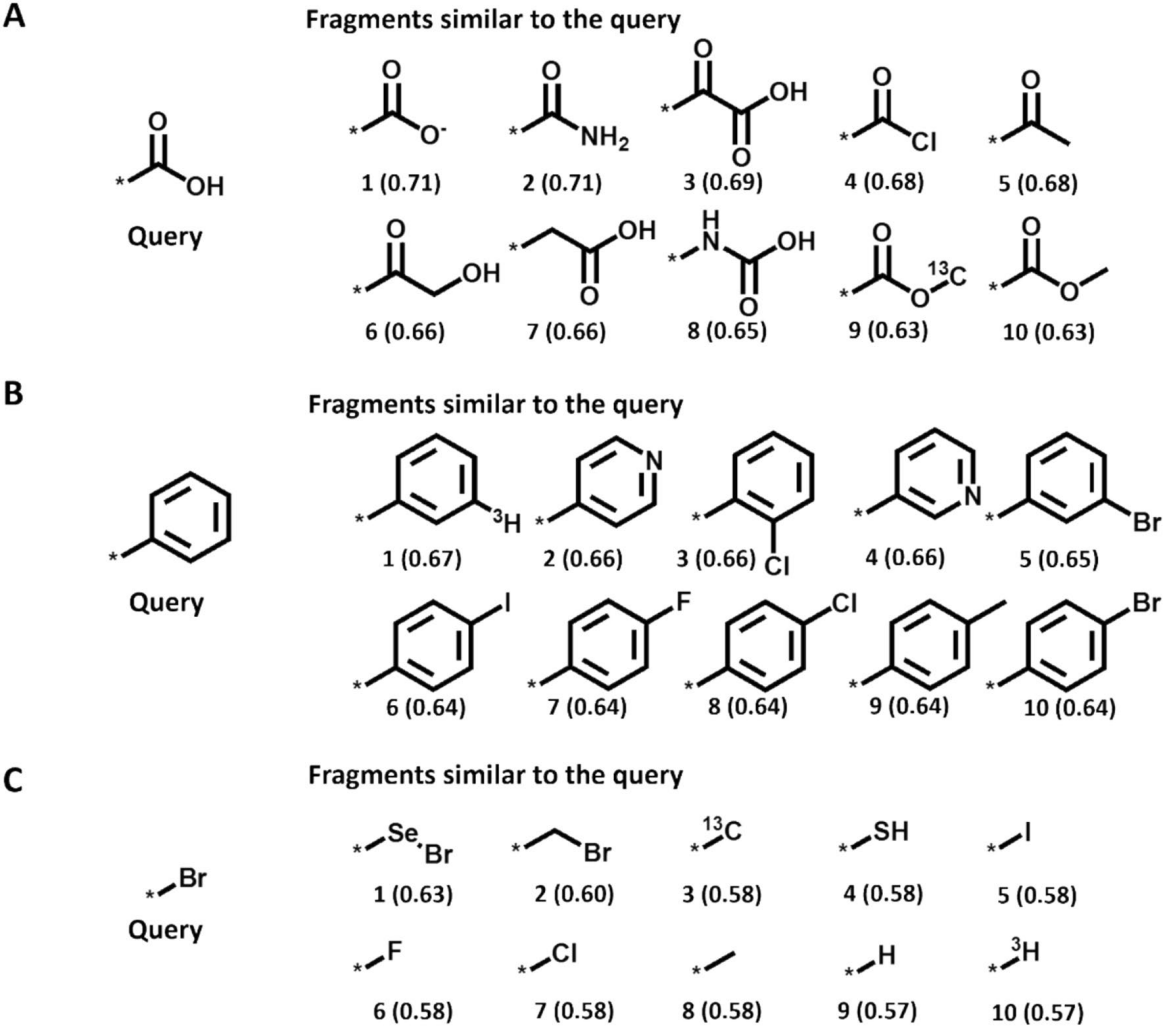
Descriptor-based fragment similarity searching. For the **A** carboxy, **B** phenyl, and **C** bromine query, the top 10 most similar fragments based on CFR similarity are shown (similarity values are reported in parentheses)

**Fig. 5:**
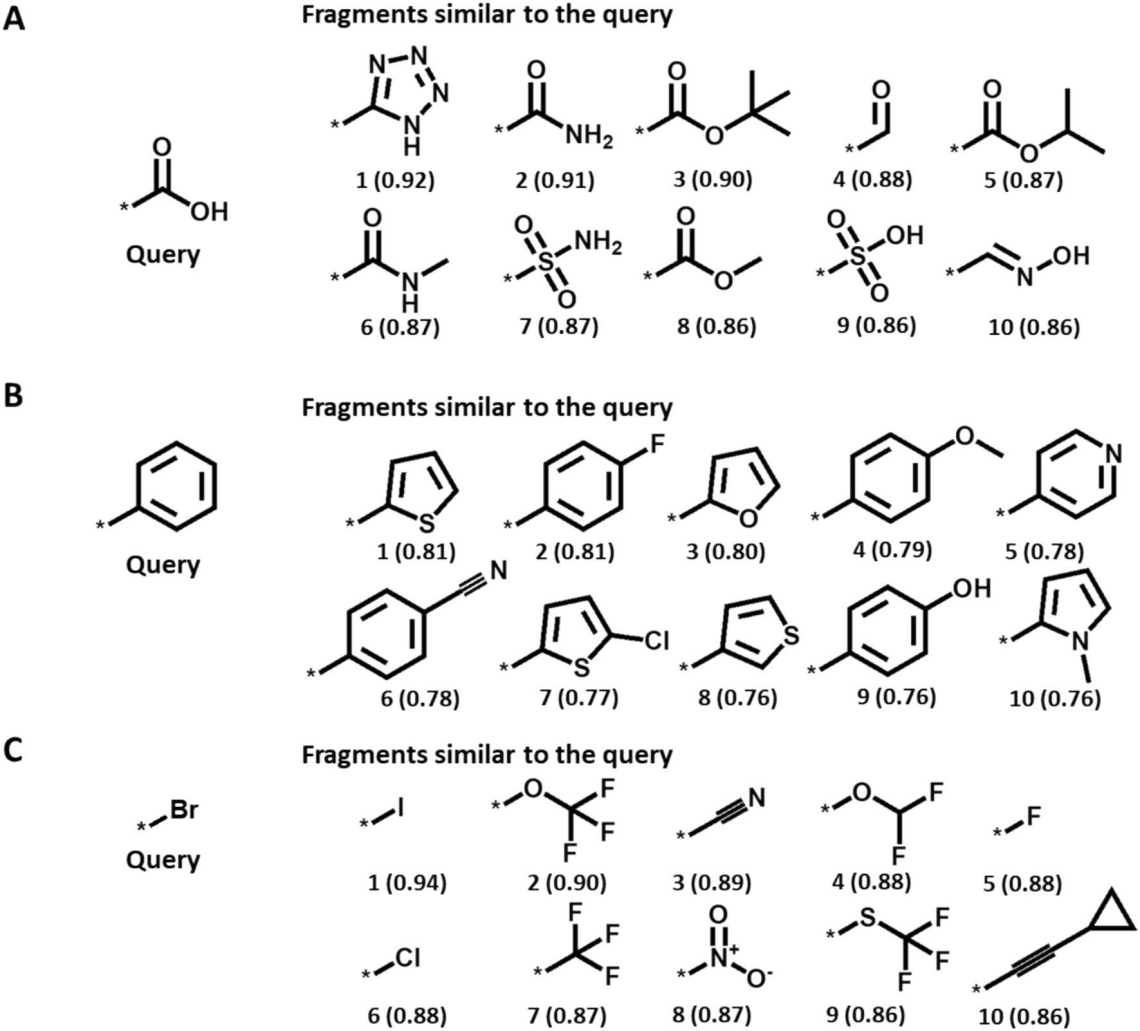
Embedded fragment vector-based similarity searching. For the **A** carboxy, **B** phenyl, and **C** bromine query, the top 10 most similar fragments based on EFV similarity are shown (similarity values are reported in parentheses)

Figures 4 and 5 show the results of CFR- and EFV-based similarity searching, respectively, for query fragments including the carboxyl group, phenyl ring, and bromo group in the database of 26,795 unique substituents. For each query, the 10 most similar fragments are shown. For the carboxyl group, all of the most similar fragments identified by CFR similarity searching were carboxyl derivatives or contained a carbonyl oxygen (Fig. 4A). However, no known bioisosteres were among the top 10 fragments in this case. For the phenyl query, CFR similarity searching identified two pyridines that are known classical bioisosteres (Fig. 4B) [31, 32]. In case of the bromine atom, other halogen substituents including chlorine (rank 7) were identified as well as the thiol group (rank 4), a classical bioisostere (Fig. 4C).

For all queries, EFV similarity searching prioritized fragments that tended to be chemically more diverse than fragments prioritized based on CFR similarity (Fig. 5). While the EFV similarity-based ranking also contained similar fragments at high ranks, chemical diversification compared to the CFR similarity-based ranking was largely due to the ability of EFV calculations to identify more non-classical bioisosteric replacements. For the carboxyl query, the 10 most similar fragments shown in Fig. 5A contained three non-classical bioisosteres including tetrazole (rank 1), sulfonamide (rank 7), and sulfonic acid (rank 9). For the phenyl query (Fig. 5B), several classical bioisosteres were identified including thiophene substituents (rank 1 and 8), furan (rank 3), and pyridine (rank 5) [31, 33]. For the bromine query (Fig. 5C), the classical chlorine bioisostere (rank 6) and two non-classical bioisosteres were prioritized based on EFV similarity including the cyano (rank 3) and trifluoromethyl group (rank 7) [31, 32]. Notably, the trifluoromethyl group and nitro group (rank 8) have also been classified as bioisosteres [34].

Taken together, the results of bioisostere searching revealed that EFV similarity prioritized more bioisostere relationships than descriptor-based CFR similarity including the identification of non-classical bioisosteres, which were not detected by CFR calculations. This was an encouraging finding that we attributed to the EFV-dependent similarity context that was absent in pairwise CFR similarity calculations.

### Global embedded fragment vector similarity

**Fig. 6:**
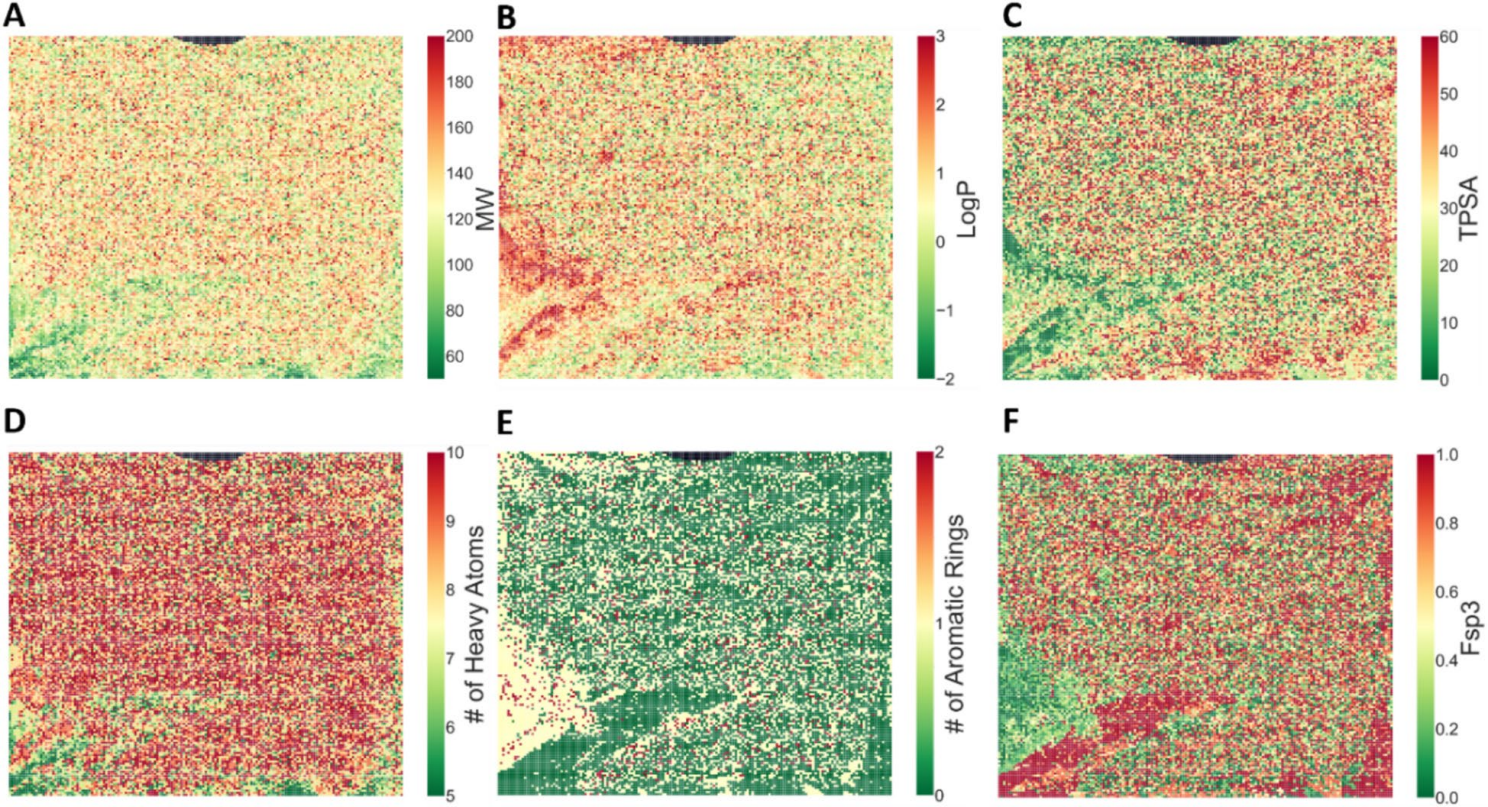
Molecular grid map. EFVs of the 26,795 substituent fragments are projected onto an MGM (of size 165 × 163 cells). EFVs are represented by cells that are color-coded according to different calculated fragment properties including **A** MW, **B** LogP, **C** TPSA, **D** number of heavy atoms, **E** number of aromatic rings, and **F** Fsp3. The small black region at the top of the MGM consists of grid points to which no fragments were assigned. “#” means “number of”

To assess EFV similarity-property relationships on a global scale, EFVs were computed using CBOW_W2V for all 26,795 substituent fragments and projected onto an MGM (see Concepts and Methods). Then, different properties including molecular weight (MW), the logarithmic octanol–water partition coefficient (LogP), topological polar surface area (TPSA), the number of heavy atoms, number of aromatic rings, and fraction of sp^3^ carbon atoms (Fsp3) were calculated for all substituents and separately mapped onto MGM cells containing their respective EFVs. The resulting MGM property distributions shown in Fig. 6 make it possible to globally assess EFV similarity-property relationships, taking into account that proximity of MGM cells provides an alternative measure of EFV similarity.

The property distributions revealed clear trends. Fragments with low MW (Fig. 6A) corresponding to a small number of heavy atoms (Fig. 6D) mainly mapped to the lower left of the MGM. In addition, fragment having high LogP (Fig. 6B) and low TPSA (Fig. 6C) values (indicating hydrophobicity) were generally located in the left half of the map. Furthermore, fragments with high Fsp3 values (Fig. 6F) accumulated in regions where the number of aromatic ring rings was small (Fig. 6E). Hence, the global MGM view revealed complementary property-based clustering of EFVs, reflecting the presence of global EFV similarity-property relationships.

### Query design for compound optimization based on word pair relationships

**Fig. 7:**
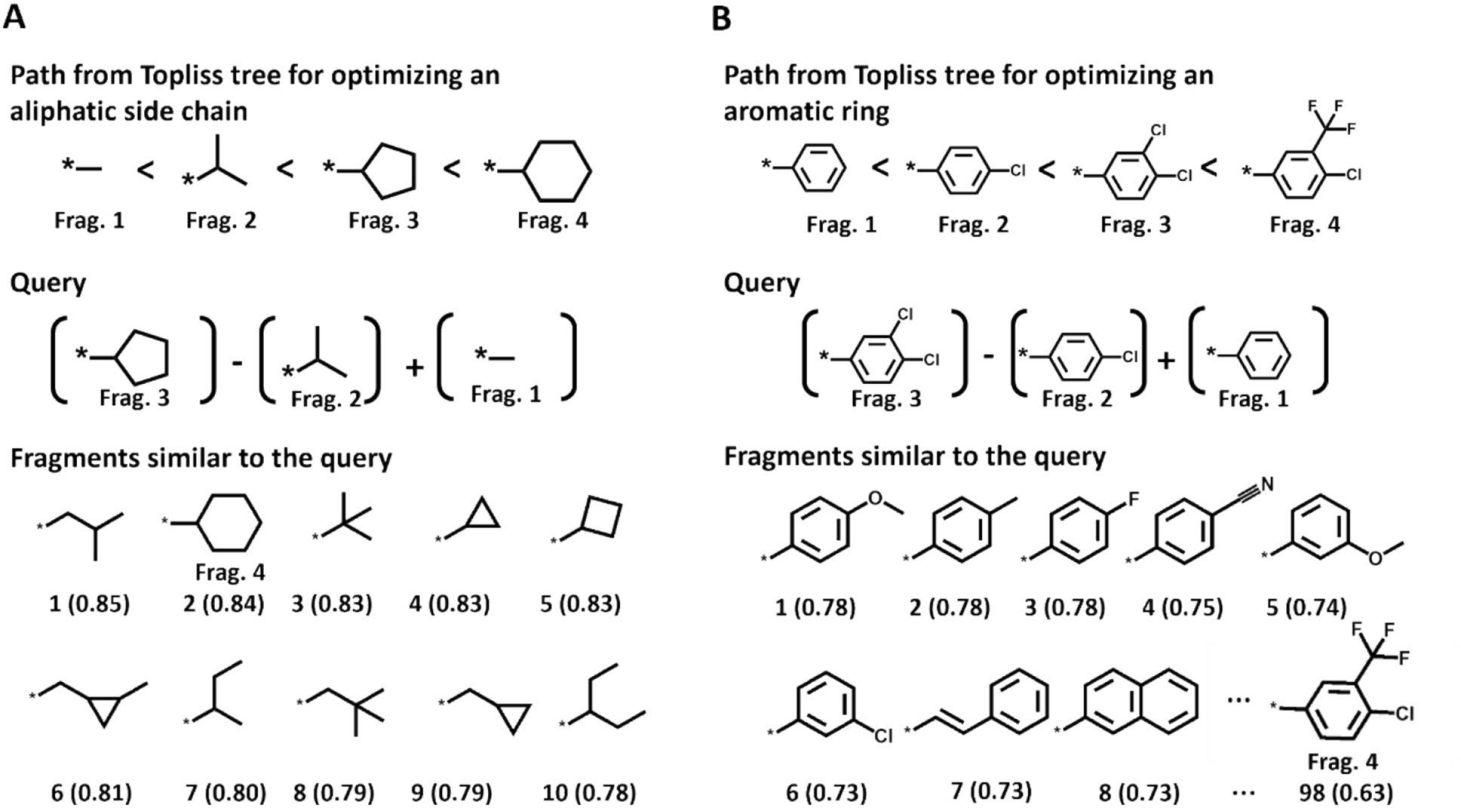
Prediction of substituents from Topliss trees. In **A** and **B**, an optimization path from the TT for aliphatic and aromatic substituents is shown, respectively, which corresponds to AS with an ascending potency gradient. In addition, for each path, a query for predicting the terminal fragment 4 based on word pair relationships and context-dependent EFV similarity and the resulting top 10 fragments most similar to the query are shown. EFV similarity values are reported in parentheses

The Topliss tree (TT) is a seminal and chemically intuitive data structure to guide compound optimization [35]. In a TT, aliphatic side chains or aromatic rings are modified in a stepwise manner to alter hydrophobicity, electronic, and steric properties in order to increase compound potency [35]. At each level in a TT, branch points define pathways of stepwise substituent modifications depending on whether a particular substituent at the branch point increases, decreases, or retains the potency of an analogue compared to the one with the preceding substituent. Therefore, the TT structure was designed to provide practical guidance for the optimization of aliphatic or aromatic substituents, suggesting the next analogue to be synthesized [35]. From TTs, optimization paths constituted by substituent sequences can be extracted that represent AS with wildcard cores and stepwise increasing potency. Such paths implicitly represent SAR transfer series and thus provide excellent test cases for the ability of CBOW_W2V to predict potent analogues, as shown in Fig. 7.

In Fig. 7A, an optimization path from the TT of aliphatic substituents is depicted, in which potency increases in the order of a methyl (fragment 1) to iso-propyl (2), cyclopentyl (3), and cyclohexyl group (4). To predict the next fragment of a sequence in a context-dependent manner, a word pair relationship-based query using EFVs can be generated, as discussed above. For example, fragment 4 can be predicted based on the preceding fragments as follows: (fragment 3 – fragment 2) + fragment 1 = fragment 4.

Accordingly, fragment 4 is predicted here using fragment 1 and the relationship between fragment 3 and 2 (captured by the difference of their EFVs). The corresponding calculation was carried out and, as shown in Fig. 7A, fragment 4 was at position 2 in the similarity-based ranking of all 26,795 substituents. An analogous prediction using the same query was performed for an optimization path of an aromatic ring in the order of the phenyl (fragment 1), 4-chlorophenyl (2), 3,4-dichlorophenyl, (3), and 4-chloro-3-(trifluoromethyl)phenyl group (4), as depicted in Fig. 7B. In this case, many similar aromatic ring substituents were prioritized at high rank positions and fragment 4 was ranked 98th of all 26,795 substituents (Fig. 7B). The lower rank compared to the aliphatic substituent was a consequence of the wealth of available similar aromatic ring substituents that had similar EFV values compared to the query. Hence, the corresponding rank positions were distinguished by small differences in EFV values and likely included other active substituents, in addition to the known fragment 4. These examples further indicate the potential of EFV operations to predict attractive substituents for extending AS during compound optimization. Generally, highly-ranked substituents in EFV similarity-based rankings are primary candidates for selection (while the entire ranking of all recorded substituents is of less interest in this case).

### Analogue series alignments

**Fig. 8:**
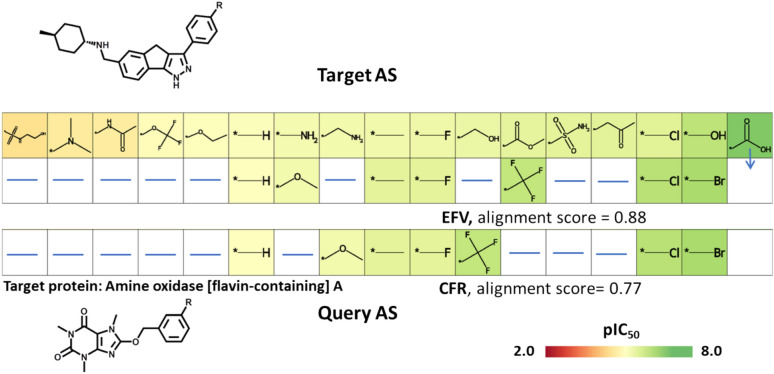
Exemplary alignment comparison. Shown are top-scoring alignments for a query AS based on EFV and CFR similarity, respectively. Alignments scores are reported and the potency of analogues is color-coded according to the pIC_50_ scale shown at the lower right

Finally, we examined and compared CFR and EFV similarity-based AS alignments resulting from search calculations using query AS. Since alignment scores based on EFV and CFR similarity are not directly comparable, we initially compared top-scoring EFV- and CFR-based alignments for individual query AS. Figure 8 shows an example of top-ranked alignments for a query AS consisting of seven analogues. These two alignments were similar and differed in two positions.

**Fig. 9:**
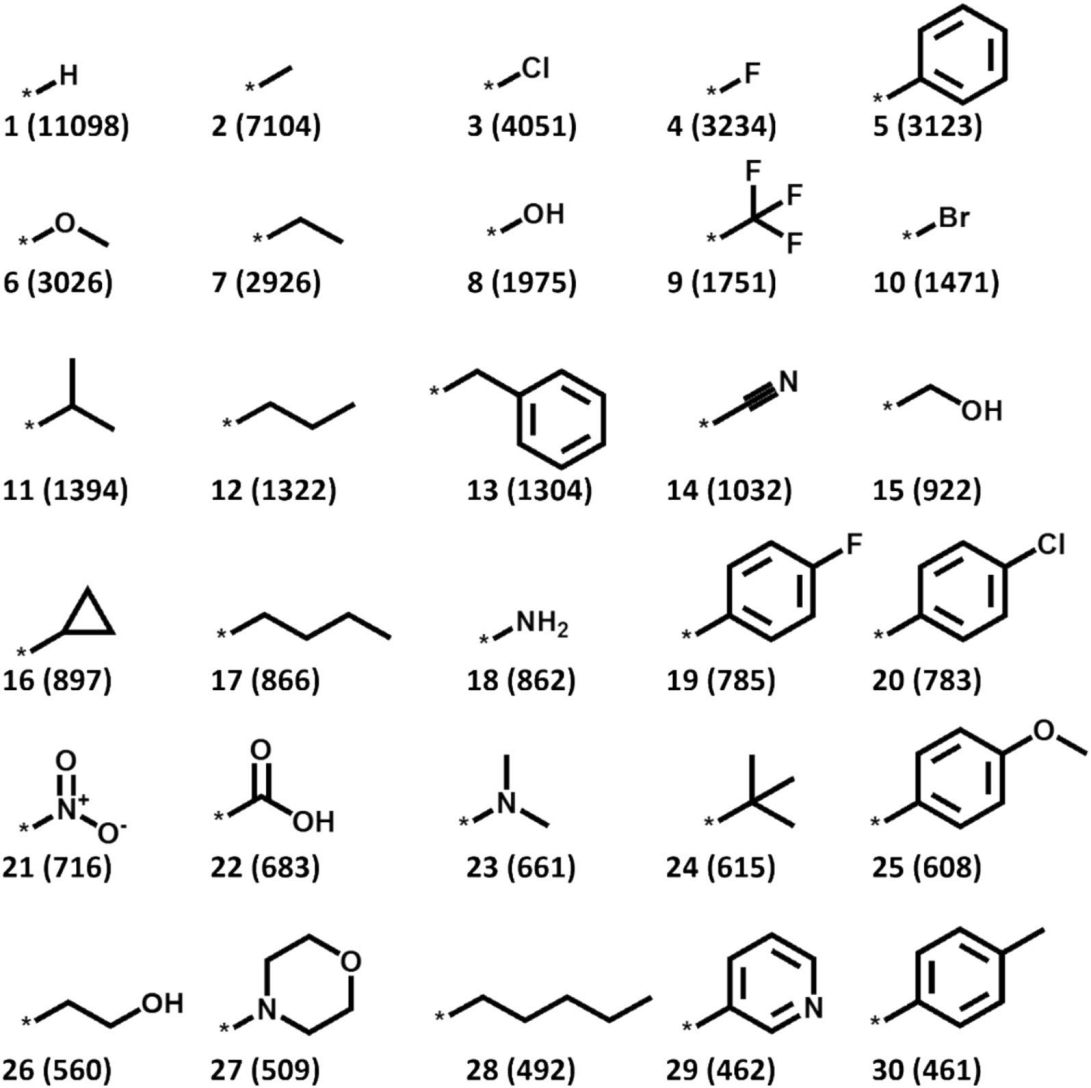
Most frequently occurring terminal substituents. Show are the top-30 most frequently occurring terminal substituents in AS. For each substituent, the number of AS in which it was found at the terminal position is reported in parentheses

To further explore pairings of bioisosteres in AS alignments, we determined the terminal substituents (corresponding to the most potent analogue) of our 113,113 potency-ordered source AS. Figure 9 shows a frequency-based ranking of terminal substituents. Many of the most frequent terminal substituents were small functional groups, for which searching for bioisosteres was not of primary interest. The most frequent substituents also included a number of phenyl ring derivatives, as one might expect. However, the ranking contained several substituents that were considered interesting candidates for the identification of bioisoteric replacements, as further discussed below.

The carboxyl group was one of the most frequent terminal substituents, found in 683 AS and ranked at position 22 (Fig. 9). For investigating replacements of the carboxyl group, we randomly selected 100 of the AS containing a terminal carboxyl group as queries, searched these query AS against our database excluding AS with carboxyl groups (110,246 AS) based on EFV and CFR similarity, respectively, and determined the number of AS alignments, in which the terminal carboxyl group was aligned with one of 13 known non-classical bioisosteres [31, 32] and/or in which a transfer analogue represented one of these bioisosteres.

**Fig. 10:**
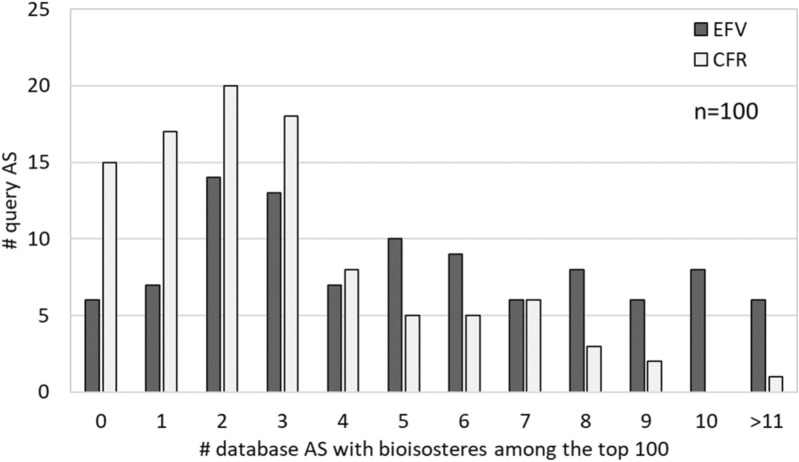
Alignments of query analogue series with a terminal carboxyl substituent. The histogram shows alignment statistics for 100 query AS with a terminal carboxyl substituent. Reported are the numbers of query AS (y-axis), for which increasing numbers (0 to 11 or more) of qualifying alignments with database AS were obtained among the 100 top-ranked alignments (x-axis) containing a non-classical bioisostere that matched the carboxyl group or represented SAR transfer analogue

Figure 10 reports the number of query AS for which increasing numbers of alignments with database AS were detected among the 100 top-scoring alignments that contained correctly aligned bioisosteres of the carboxyl group. Search calculations based on EFV similarity produced many more qualifying alignments than CFR-based calculations, hence reinforcing the use of context-dependent similarity for systematic AS alignments.

**Fig. 11:**
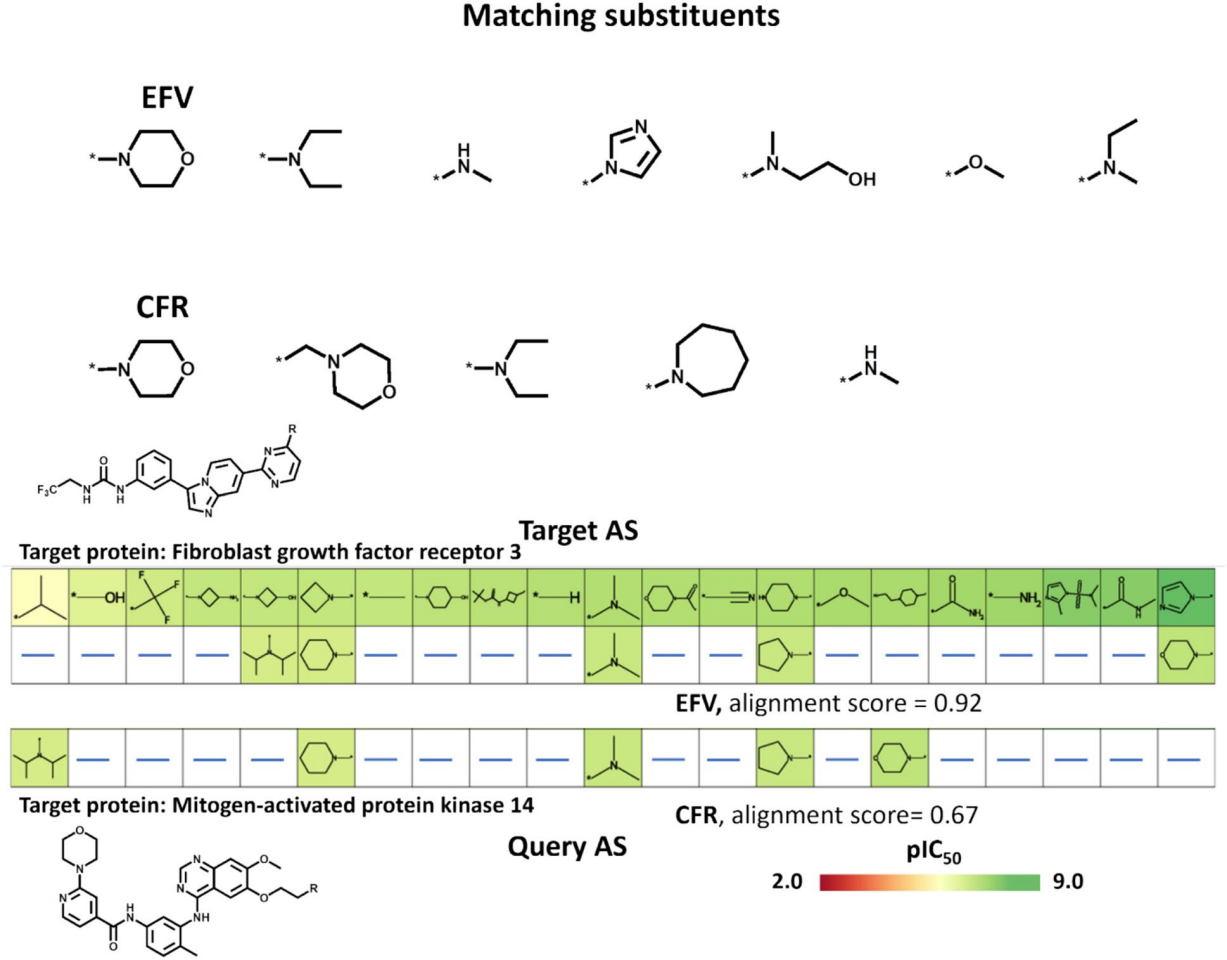
Alignments for a query series with terminal morpholine substituent. At the top, morpholine-matching substituents identified in AS alignments based on EFV and CRF similarity are shown, respectively. At the bottom, high-scoring alignments for the query AS are shown based on EFV and CFR similarity, respectively. Alignments scores are reported and the potency of analogues is color-coded according to the pIC_50_ scale shown at the lower right

As another example, we investigated alignments for a query AS with a terminal morpholine substituent, ranked 27th (Fig. 9). Alignments based on EFV and CRF similarity identified seven and five matching substituents (including morpholine), respectively, as shown in Fig. 11. The morpholine-matching substituents identified based on EFV included the imidazole bioisostere that was not dectected based on CRF. Figure 11 also shows highly scoring alignments for the query AS with terminal morpholine substituent. In the EFV-based alignment, imidazole was detected as a morpholine-matching substituent.

## Conclusion

SAR transfer events across different targets are a topical issue in medicinal chemistry. Commonly applied chemical strategies to optimize compounds for different (hydrophobic or polar) ligand-target interactions frequently lead to comparable SAR progression of different AS with activtiy against the same or different targets. Therefore, SAR transfer provides a valuable source of knowledge for generating AS with sustainable SAR features. SAR transfer events are best identifed through systematic exploration of AS alignments. In this work, we have introduced a second-generation methodology for AS database searching and alignment that combines dynamic programming with context-dependent similarity calculations adapted from the assessment of word similarity in NLP, facilitated through the Word2vec approach. Key features of the resulting CBOW_W2V methodology reported herein for the detection of SAR transfer events include its ability to detect non-classical bioisosteres for functional groups and capture substituent-property relationships on a global scale. These features are attributable to the novel context-dependent assessment of substituent similarity, setting it apart from conventional molecular descriptor-based similarity measures. For small structural fragments such as substituents, descriptor-based similarity assessment has intrinsic limitations, given the small number of detectable features and confined property differences between many small substituents. These limitations are mirrored by the restricted ability of CFR calculations to detect bioisosteres with structural variations. For AS alignments relying on the assessment of substituent similarity and similarity-activity relationships such as those of SAR transfer AS, comprehensively accounting for bioisostere pairings is of critical relevance. To this end, context-based similarity assessment reveals a clear advantage over conventional CFR similarity, rendering the methodology introduced in our current study particularly attractive for generating AS alignments in the search for SAR transfer events. Furthermore, context-depending similarity assessment, as reported herein, is conceptually novel in computational medicinal chemistry and should thus also be of more general relevance for other applications.

## Data Availability

The CBOW_W2V method including source code and the large AS database generated for our analysis are available via the following link: https://uni-bonn.sciebo.de/s/Y9vWFfog272Mno6.

## Abbreviations

AS: Analogues series
CBOW: Continuous bag of words
CBOW_W2V: CBOW Word2vec
CFR: Conventional fragment representation
EFV: Embedded fragment vector
FP: Fingerprint
Fsp3: Fraction of sp^3^ carbon atoms
J-V: Jonker-Volgenant
LogP: Logarithmic octanol–water partition coefficient
MGM: Molecular grid map
MMP: Matched molecular pair
MQN: Molecular quantum number
MW: Molecular weight
NLP: Natural language processing
PCA: Principal component analysis
SAR: Structure–activity relationship
TPSA: Topological polar surface area
t-SNE: T-distributed stochastic neighbor embedding
TT: Topliss tree
